# Surface Characterization and Corrosion Resistance of Biomedical AZ31 Mg Alloy Treated by Microarc Fluorination

**DOI:** 10.1155/2020/5936789

**Published:** 2020-10-27

**Authors:** Lin Sun, Bing Cheng Zhao, Teng Wang, Jia Yi Cui, ShuXin Zhang, Feng Li, Qianqian Zhang, HongXin Cai, Heng Bo Jiang, Eui-Seok Lee

**Affiliations:** ^1^Stomatological Materials Laboratory, School of Stomatology, Shandong First Medical University & Shandong Academy of Medical Sciences, Tai'an, Shandong 271016, China; ^2^Shandong Liming Institute of Technology and Vocational College, Tai'an, Shandong 271000, China; ^3^Department of Oral and Maxillofacial Surgery, Graduate School of Clinical Dentistry, Korea University, Seoul 08308, Republic of Korea

## Abstract

The application prospect of biodegradable materials is being studied extensively. However, the high corrosion rate and its alloys in body fluids have been major limitations of the application of pure Mg (magnesium). To improve corrosion resistance of biodegradable AZ31 Mg alloy, we adopted microarc fluorination within a voltage range of 100-300 V in 46% hydrofluoric acid. To obtain morphologies, chemical compositions, and structural characteristics, field-emission scanning electron microscopy (FE-SEM), energy-dispersive X-ray spectroscopy (EDS), and X-ray diffraction (XRD) were performed, respectively. Results showed that the coating was mainly composed of MgF_2_. Electrochemical corrosion and immersion tests proved that the corrosion resistance of MAF-treated AZ31 Mg alloy was significantly improved compared with untreated AZ31 Mg alloy in HBSS (Hank's Balanced Salt Solution). Current densities of AZ31, MAF100, MAF150, MAF200, MAF250, and MAF300 were 342.4, 0.295, 0.228, 0.177, 0.199, and 0.212 *μ*A/cm^2^, respectively. The roughness test indicated that samples under MAF treatment of 200 V, 250 V, and 300 V had large surface roughness. Meanwhile, the contact angle measurement and surface free energy test suggested that those samples had smaller contact angle and higher SFE than Ti. Thus, MAF-treated AZ31 Mg alloy might have promising application in various fields.

## 1. Introduction

In orthodontic treatment of oral cavity, microimplant nails are often used in anchorage treatment. Screws must be removed from patients after treatment. During the removal of screws, there is a risk of screw breakage. The broken screw might remain in a patient due to an operational error [1]. Removal of the broken screw might cause secondary injury to the patient. If the screw is made of biodegradable material, the broken portion of the screw can naturally degrade in the patient. Mg has been a research focus in recent years [2, 3]. Compared to other biodegradable materials, Mg and its alloys have higher specific strength, better degradation ability, and better seismic performance [4, 5]. For example, the elastic modulus of Mg or its alloy (45 GPa) is close to that of the human bone (20 GPa), thus alleviating stress shielding effect between the bone and implant. However, the elastic modulus is about 110 GPa for titanium alloy and about 200 GPa for stainless steel, making it easier to cause the stress shielding effect. Besides, the density of metallic magnesium is 1.7-2.0 g/cm^3^, similar to the density (1.8-2.1 g/cm^3^) of a natural bone. These characteristics indicate that Mg and its alloy are promising bone implant materials [6]. However, their degradation rate is too high to produce microimplant nails. Therefore, Mg and its alloys need to have an appropriate degradation rate to ensure their mechanical properties and normal metabolism of hydrogen.

Our main research direction is to slow down the degradation rate of Mg in vivo. Currently, degradable membrane formed on the metal surface through metal passivation treatment, metal plating [7, 8], or metal surface fluorination [9] can delay the degradation of metal. These methods can maintain good mechanical properties [10, 11]. At present, electrochemical methods have been widely used for surface treatment of Mg. These methods have been proven to be extremely effective [12–14].

Microarc oxidation (MAO) is a surface treatment method carried out under high voltage. The coating prepared by MAO has advantages of short preparation time, uniform surface characteristics similar to the bone, adjustable coating thickness, and high biocompatibility. Properties of MAO coatings are mainly determined by electrolytes used, including borate [15], silicate [16], fluoride [13], potassium fluorozirconate [17], phosphate [18], and aluminate [19]. Coating surface morphology and thickness are main factors affecting MAO coatings [20]. For example, the porosity of MAO coating is decreased with an improvement of frequency, but increased with an increase of the final voltage. Through pores and microcracks in MAO coatings lead to electrochemical corrosion of Mg alloys more easily than nonthrough pores [21]. Sealing is an important method to improve the physical barrier role of MAO coating. The coating can be sealed using silicates, phosphates, sol gels, and alkaline materials with additional polymer coatings [22, 23].

Fluorine (F) is an essential trace element. A proper amount of fluorine is beneficial for the human health [24]. MgF_2_ coating is formed by infiltrating Mg in hydrofluoric acid for more than 24 hours. However, the biocompatibility of the MgF_2_ coating is poor [13]. That MgF_2_ coating also has disadvantages of long preparation time and environmental pollution [9, 25]. A high F content in MAO coating is toxic. A recent study has shown that MAO coating containing high F content (higher than 19 at%) exhibits high toxicity [26]. One study has shown that MAO coating containing the F content of 62.12 at % can improve the proliferation of cells [13].

To improve corrosion resistance of Mg, we adopted the method of microarc fluorination (MAF), a combination of MAO and fluorination. The coating prepared by MAF overcomes disadvantages of MAO coating's easy cracking and smooth surface prepared by fluorination [13].

In this study, hydrofluoric acid was used as an electrolyte to prepare coating at a voltage range of 100-300 V. Then, the coatings were immersed in Hank's Balanced Salt Solution (HBSS) for corrosion experiment. After the corrosion experiment, surface analysis was carried out using field-emission scanning electron microscope (FE-SEM), energy-dispersive X-ray spectroscopy (EDS), and X-ray diffraction (XRD). Roughness, contact angle, and surface free energy (SFE) of the corroded sample were measured. Corrosion resistances of samples were analyzed.

Most experiments on the corrosion performance of Mg are in vitro experiments [27–29]. Few experiments have explained the corrosion behavior of Mg in vivo. We used HBSS to conduct the immersion experiment to simulate an in vivo experiment. Limited by ethical aspects and laboratory conditions, it is impossible to use animal models to simulate in vivo experiments.

## 2. Materials and Methods

### 2.1. Sample Preparations

AZ31 Mg alloy discs (diameter = 16 mm; thickness = 2 mm) were laser cut from the AZ31 sheet. Its compositions are shown in Table 1. Titanium discs (diameter = 16 mm; thickness = 2 mm) were prepared as references for surface free energy analysis. Samples were polished with a 2000# SiC sandpaper in absolute ethyl alcohol, washed with absolute ethyl alcohol, and blow-dried.

### 2.2. MAF Treatment

**Figure 1:**
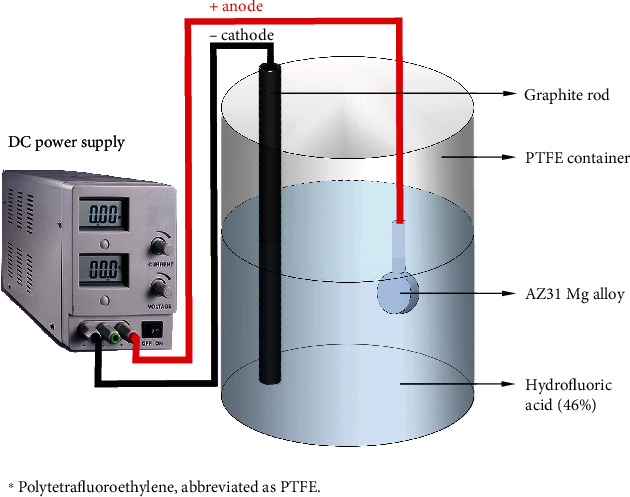
Diagram of the MAF treatment of AZ31 Mg alloy.

In the electrolyte of 100 mL hydrofluoric acid (Sigma-Aldrich, 46%), AZ31 Mg alloy was used as an anode, and a graphite rod was used as a cathode. After numerous experiments, it was found that a large amount of hydrofluoric acid gas was released during a violent reaction of hydrofluoric acid at a high voltage, polluting the environment and harming health. Therefore, 100, 150, 200, 250, and 300 V were chosen as experimental voltages in the present study. Five sets of different voltages (100, 150, 200, 250, and 300) were applied at a DC constant voltage with a maximum current of 2 A for 30 seconds. MAF-treated AZ31 Mg alloy was then prepared and rinsed with deionized water three times and blow-dried. A diagram of the MAF process is shown in Figure 1. The code name and the process of preparing MAF-treated samples are shown in Table 2.

### 2.3. Surface Characterization

Surface morphology and basic elements of samples were observed using an FE-SEM (JSM-7001F; JEOL, Ltd, Tokyo, Japan) and EDS. Surface compositions of samples were determined by XRD. At 40 kV and 30 mA, the scan rate was 1°/min using a Cu-K*α* line.

Surface roughness was measured by the 3D optical profilometry (ContourGT, Bruker, Tucson, AZ, USA). The scattering angle was fixed at a smaller value of 1. According to a single diffuse scattering scan, Ra, Rp, Rq, Rt, and Rv values were measured based on ISO 4287 standard. The sampling area was 0.17 × 0.2 mm^2^.

### 2.4. Surface Free Energy Analysis

Deionized water and di-iodomethane as test liquids were used for contact angle measurements. A drop of test liquid (5 *μ*L droplet from a syringe) was placed on the surface of each sample for 60 s. The equilibrium contact angle was then determined with an image analyzer. The contact angle was recorded for both sides. The SFE of each sample was calculated using Eq. (1) and Eq. (2).

(1) $$\begin{array}{c} \gamma =\gamma^{p}+\gamma^{d}, \end{array}$$

(2) $$\begin{array}{c} \left(1+\cos\theta \right)\gamma_{1}=2{\left({\gamma_{s}}^{d}{\gamma_{1}}^{d}\right)}^{1/2}+2{\left({\gamma_{s}}^{p}{\gamma_{1}}^{p}\right)}^{1/2}, \end{array}$$

where *θ* is the mean equilibrium contact angle of H_2_O or CH_2_I_2_, *γ*_l_ is the surface free energy of water, *γ*_l_^*d*^ and *γ*_l_^*p*^ refer to the hydrogen bonding and dispersion force components of *γ*_l_; *γ*_*s*_ is the surface free energy of samples, and *γ*_*s*_^*d*^ and *γ*_*s*_^*p*^ refer to the dipole-dipole interactions and dispersion force components of *γ*_*s*_.

### 2.5. Electrochemical Corrosion Tests

Corrosion resistances of AZ31 and MAF-treated AZ31 Mg alloys were determined with the open circuit potential and potentiodynamic polarization test. An Ag/AgCl/Sat-KCl electrode was used as a reference electrode. A pure graphite rod was used as a reaction electrode. Each sample was exposed (1 cm^2^) as a working electrode, and 1000 mL of HBSS (Shanghai Yuanye Bio-Technology Co, Ltd, China) was used as an electrolyte. The temperature of the electrolyte was maintained at 37 ± 1°C. In this test, in order to stabilize the potential of the test sample, the sample was first immersed in PBS for 1 hour to perform its open circuit potential (OCP) mode. Then, a potential dynamic polarization (PDP) test was performed. Electrochemical tests were performed using a VersaSTAT3 potentiostat (Model 300, AMETEK, Inc, Berwyn, PA, USA) coupled with a VersaStudio 2.43.3 software (AMETEK, Inc.) for electrochemical control and data analyses.

### 2.6. Immersion Corrosion Tests

AZ31 and MAF-treated AZ31 Mg alloys were vertically immersed in HBSS at 37°C for one week and four weeks. Each group had three parallel samples. The ratio of the HBSS volume to the sample area was 20 mL/cm^2^. HBSS was refreshed weekly. After immersion for one week or four weeks, samples were ultrasonically cleaned with a chromium trioxide (CrO_3_) solution (Aladdin Inc. China) for 3 minutes, rinsed with deionized water three times, and blow-dried. The percentage of mass loss was calculated using Eq. [3]:

Mass loss% = (*M*O − *M*1/*M*0) × 100%, [3]where *M*_0_ is the mass of samples before immersion. *M*_1_ is the mass of the samples after the immersion. Three samples in each group were tested, and mass loss was expressed as mean standard deviation (SD).

### 2.7. Statistical Analysis

All data are expressed as mean ± standard deviation. Data of the materials were subjected to one-way analysis of variance (ANOVA) and post-hoc analysis using Tukey's test. Student's test was used to compare values before and after MAF treatment. All statistical analysis was performed using IBM SPSS Statistics version 23.0 for Windows (IBM Corporation, Armonk, New York, USA). Statistical significance was defined at *p* < 0.01.

## 3. Results and Discussion

**Figure 2:**
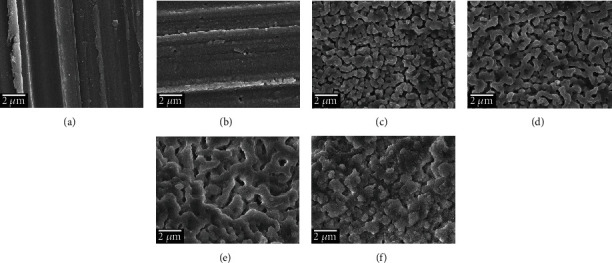
FE-SEM surface morphology of (a) AZ31, (b) MAF100, (c) MAF150, (d) MAF200, (e) MAF250, and (f) MAF300.

Figure 2 displays surface topographies of AZ31 and MAF-treated AZ31 Mg alloy. The surface of MAF100 was not smooth. There were several small holes and particles. The coral-like structure appeared densely from 150 V but disappeared at 250 V. Diameters of crystal particles for MAF150, MAF200, and MAF250 were approximately 0.5, 0.6, and 0.7 *μ*m, respectively. The coral-like structure was gradually fused, forming micropores that could promote adhesion and proliferation of osteoblasts [30]. However, MAF300 crystal particles were nonuniform in diameter.

**Figure 3:**
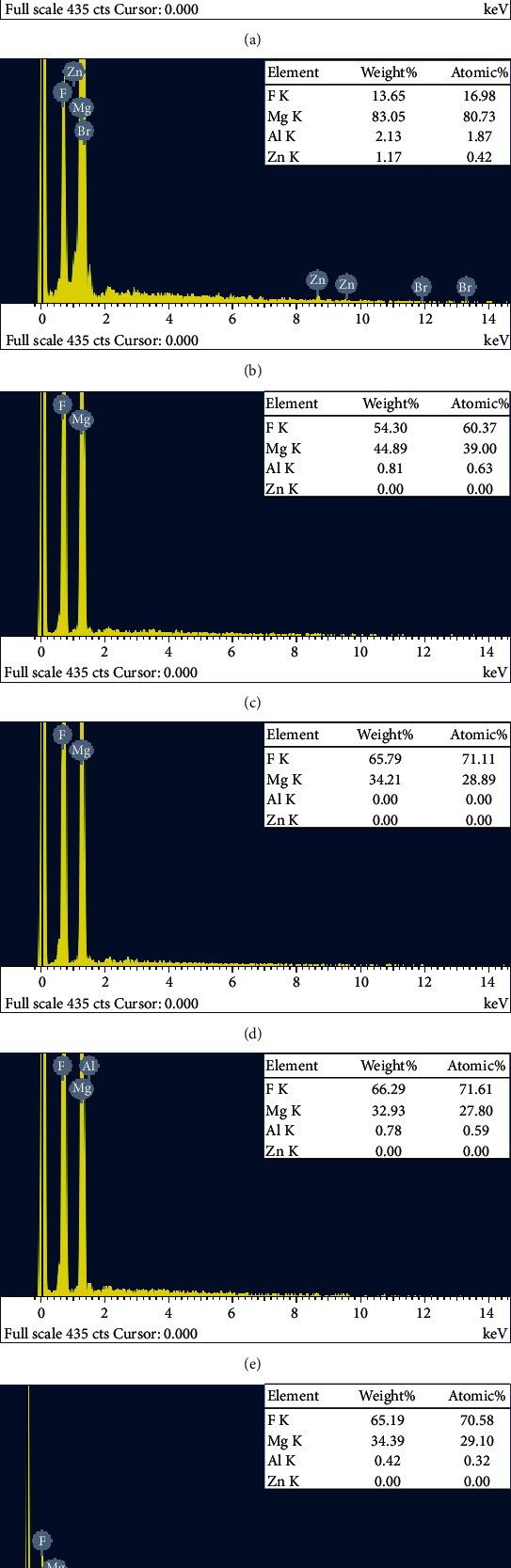
EDS analysis of (a) AZ31, (b) MAF100, (c) MAF150, (d) MAF200, (e) MAF250, and (f) MAF300.

Figure 3 demonstrates that surface elements are mainly composed of F and Mg. Thus, it could be concluded that magnesium fluoride coatings were formed on surfaces of the samples through MAF. Based on chemical element compositions on surfaces of AZ31, MAF100, MAF150, MAF200, MAF250, and MAF300 based on EDS analysis, the percentage of fluoride increased within increasing voltage. Compared with MAF100, MAF150 exhibited a significant increase of fluoride, achieving an increase of 43.39%. Fluoride percentages on surface of MAF200, MAF250, and MAF300 were 71.11%, 71.61%, and 70.58%, respectively. At 150 V, The AZ31 Mg alloy surface appeared as an obvious spark discharge phenomenon. However, with further increase of voltage, the strong spark discharge deposition in the anodic oxidation process of AZ31 Mg alloy released a large amount of heat, resulting in overheating of the surface of the AZ31 Mg alloy and a significant increase of the temperature of local solution which accelerated the dissolution rate of the oxide film. Therefore, coating after 150 V caused little change in the fluorine content.

**Figure 4:**
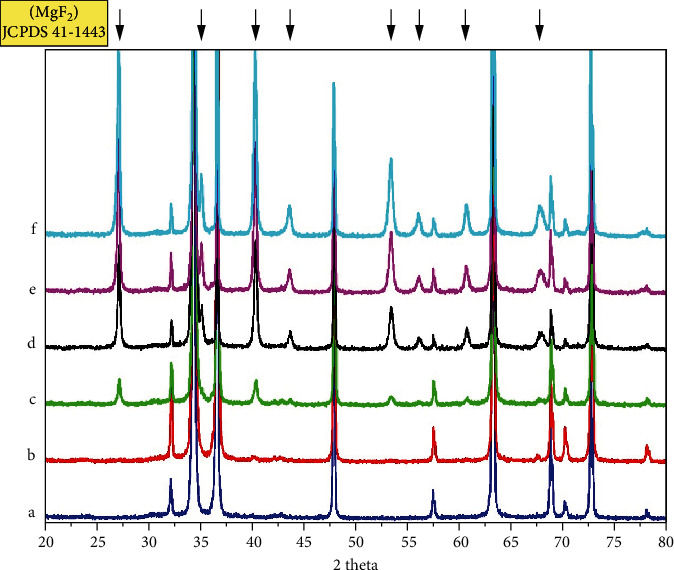
XRD patterns of (a) AZ31, (b) MAF100, (c) MAF150, (d) MAF200, (e) MAF250, and (f) MAF300.

Figure 4 presents crystalline structures of samples by XRD analysis. Referring to the standard JCPDS card, the coating was mainly made up of tetragonal MgF_2_ (JCPDS No. 411443). Compared with untreated AZ31, patterns observed for MAF-treated AZ31 Mg alloys clearly indicated the presence of tetragonal MgF_2_. As shown in Figure 4, MAF150, MAF200, MAF250, and MAF300 basically displayed the same number of diffraction peaks, angular position, relative intensity order, and shape of the diffraction peak, whereas MAF100 showed a difference possibly due to its thinner coating. X-ray projection requires tested substance to have a certain thickness. Thus, there were no peaks appearing in the relative diffraction angle for MAF100. The formation of the MgF_2_ phase in the coating is through microarc oxidation reaction of the AZ31 Mg alloy substrate and hydrofluoric acid solution in the discharging channel produced by sparks. During sparks, Mg^2+^ from substrate migrates outward. Meanwhile, F-inward migrates into channels owing to the electrical field. MgF_2_ coating forms through the following reactions:

(3) $$\begin{array}{c} \text{Mg}\to \text{M}{\text{g}}^{2+}+2{\text{e}}^{-},\\ \text{M}{\text{g}}^{2+}+2\text{F}\to \text{Mg}{\text{F}}_{2}. \end{array}$$

In a high-voltage electrolyte, the discharge on the AZ31 Mg alloy surface generated many electric sparks, resulting in a porous structure (Figure 2). A stable MgF_2_ coating was formed on the surface. It is increased gradually, with little or no sparks at a certain thickness of coating.

**Figure 5:**
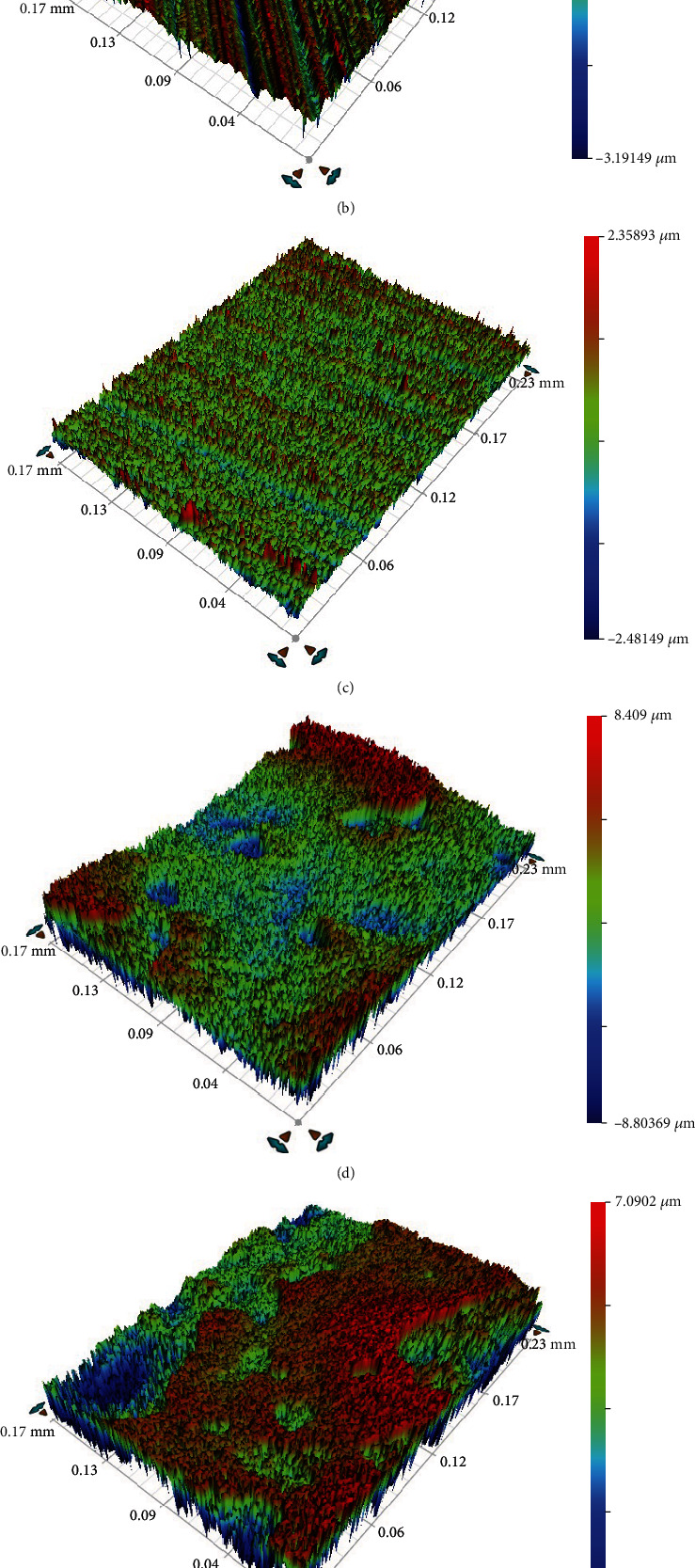
Surface roughness by 3D optical profilometry of (a) AZ31, (b) MAF100, (c) MAF150, (d) MAF200, (e) MAF250 and (f) MAF300.

Figure 5 shows surface roughness of samples by 3D optical profilometry. The image qualitatively analyzed surface roughness and visually displayed sample surface roughness. The formation of surface roughness is generally related to the processing method and other factors [31]. Due to differences in processing methods and workpiece materials, traces left on the processed surface are different in depth, density, shape, and texture [32]. The main parameter to evaluate surface roughness is contour arithmetic mean deviation-Ra which refers to the arithmetic mean value of the distance from each point on the measured contour to the reference line within the sampling range as defined in ISO 4287. As shown in Table 3, Ra values of AZ31, MAF100, MAF150, MAF250, and MAF300 were 0.182, 0.219, 0.294, 2.010, 3.235, and 6.689, respectively. Samples under treatment of MAF200, MAF250 and MAF300 had a large surface roughness. Moreover, those treated with MAF300 had significantly increased surface roughness. At a low voltage, the film growth rate was slow. However, the resulting film was dense, and the roughness was reduced. At a high voltage, the intense spark discharge deposition in the anodic oxidation process of AZ31 Mg alloy released a large amount of heat, and the electrolyte temperature increased, resulting in an increase of the dissolution rate of the film which led to an uneven film and increased roughness. There are large troughs on the surface of rough parts. They are sensitive to stress concentration such as sharp notch and cracks. Thus, surface roughness affects fatigue strengths of parts [33]. Meanwhile, rough surface can easily cause corrosive gases or liquids to penetrate into the inner layer of metal through microscopic concave valley of the surface, resulting in surface corrosion.

**Figure 6:**
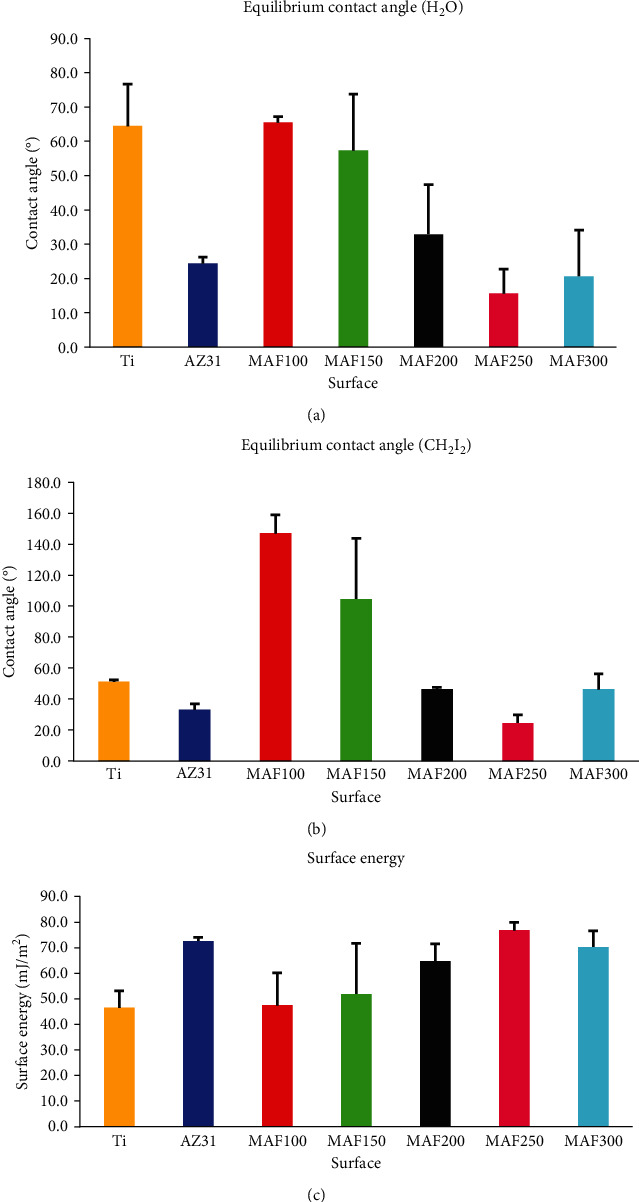
Contact angle and surface free energy of Ti, AZ31, MAF100, MAF150, MAF200, MAF250, and MAF300. Contact angle measurement with (a) H_2_O, (b) CH_2_I_2_ as probe reagent, and (c) values of surface free energy.

Figures 6(a) and 6(b) show SFE and contact angles of Ti, AZ31, and MAF-treated AZ31 Mg alloy. In clinical practice, titanium as an emerging material has been widely used in fields of surgical instruments and implants in the world. It has achieved great success. Thus, it was selected as a reference object as in previous studies [34–36]. It was found that MAF100 and MAF150 had lower SFE than other samples. Simultaneously, it could be concluded that their hydrophilicity and lipophilicity were poor as shown in Figure 6(c). Values of contact angles and SFE of them are showed in Table 4. One study has revealed that incremental SFE can result in a faster colonization of the surface, indicating excellent biocompatibility [37]. Therefore, we propose that biocompatibility of MAF100 and MAF150 is less than Ti and AZ31. Thus, MAF100 and MAF150 might be more suitable than Ti and AZ31 for fields such as bone plates that do not require good tissue compatibility. However, MAF200, MAF250, and MAF300 had higher SFE and better hydrophilicity and lipophilicity than other samples. Hence, they have the potential to be biocompatible and are more likely to be suitable than Ti for dental implants, anchorage nails, and so on that need materials to possess good tissue compatibility. FE-SEM revealed that microporous structures were formed on surfaces of AZ31 treated under high voltages which might result in an increase of the surface area. Due to capillary principle, liquid can easily infiltrate [38]. Thus, hydrophilicity and lipophilicity of MAF200, MAF250, and MAF300 were inferable to be good.

**Figure 7:**
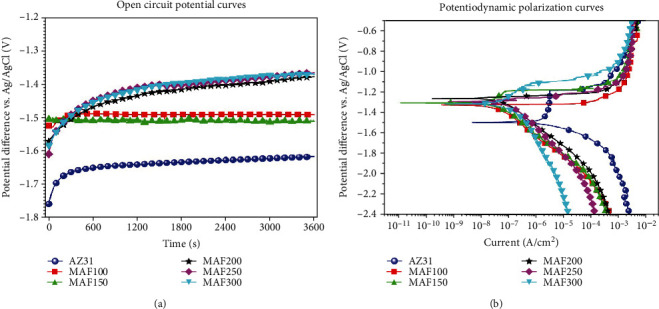
Electrochemical corrosion results. (a) OCP. (b) PDP curves of the AZ31, MAF100, MAF150, MAF200, MAF250, and MAF300.

Figure 7(a) shows OCP curves of AZ31 and MAF-treated AZ31. At 60 min, MAF200, MAF250, and MAF300 processed higher OCP values than other samples, suggesting that they had lower corrosion potential. Overall, the OCP curve tended to plateau in 60 min. In addition, the OCP values of MAF-treated AZ31 Mg alloys were improved than those of AZ31, indicating that the prepared coating had good corrosion resistance.

Figure 7(b) shows the typical Tafel curve of AZ31 and MAF-treated AZ31 Mg alloy. It was obtained by plotting *E* and log | *I*∣, where *I* was the current density and *E* was the overpotential. The combination of a higher corrosion current density and a lower corrosion potential means a higher corrosion rate [39]. From Figure 7, we can infer that with an increase in the treatment voltage of microarc fluorination, the polarization curve of the sample showed a shift to lower current density value. These results indicated that AZ31 Mg alloy exhibited a general corrosion behavior in electrolyte while the corrosion of MAF-treated Mg alloys was hindered. In addition, MAF200 had the highest corrosion potential (-1.262 V) and the lowest current density (0.177 *μ*A/cm^2^). Therefore, it is reasonable to assume that the corrosion resistance of MAF200 is the best. The results of the potentiodynamic corrosion test on AZ31 and MAF-treated AZ31 are showed in Table 5.

**Figure 8:**
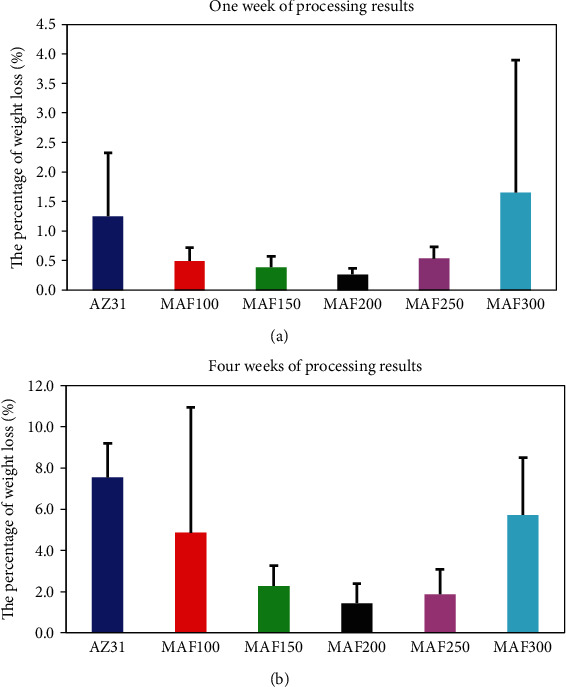
Weight loss variation over time of AZ31, MAF100, MAF150, MAF200, MAF250, and MAF300. (a) Results of one week. (b) Results of four weeks.

Figure 8 shows weight loss percentages of AZ31 and MAF-treated AZ31 Mg alloy after immersion in HBSS. In a certain voltage of 100 V and 200 V, with an increase of the treatment voltage, the weight loss of AZ31 Mg alloy decreased. The weight loss value of MAF200 was the lowest. The weight loss value of MAF250 and MAF300 was gradually increased compared with the weight loss value of MAF200, indicating that the degradation rate increased with increasing voltage. It might be related to the increase of surface roughness for MAF250 and MAF300. As confirmed in previous studies, surface roughness had a significant impact on the corrosion rate of AZ31 Mg alloy, leading to the accelerated corrosion rate with an increase of roughness [40]. Weight loss values of AZ31 and MAF-treated AZ31 exhibited basically identical trend after one week and four weeks. We could infer that corrosion resistance was increased with increasing surface roughness in several cases. In addition to roughness, the pore size simultaneously affects the protective ability of AZ31 substrate [21]. After microarc fluorination, coral-like fluorine coating was formed on the surface of AZ31 Mg alloy. It played a protective role on the surface [41]. Coating thickness and pore size are crucial to improve corrosion resistance of a sample.

## 4. Conclusions

In this study, we used MAF technology to treat and detect AZ31 in 46% hydrofluoric acid electrolyte at different voltages. We could draw the following conclusions:

1. The coral-like structure was formed on the surface by MAF
2. MAF-treated AZ31, in comparison with untreated AZ31, showed significantly improved surface roughness
3. Surface roughness increased with increasing MAF treatment voltage
4. The corrosion resistance of a sample was determined by pore size, surface roughness of the coating, and so on

## Figures and Tables

**Table 1:** Elemental compositions of AZ31 Mg alloy.

Element	Weight %	Element	Weight %
Al	2.85	Fe	0.003
Zn	0.75	Cu	0.00045
Mn	0.62	Ni	0.00052
Si	0.025	Mg	In balance

**Table 2:** Sample code name and the process of preparing MAF treatment.

Code	Surface treatment	Processing time
AZ31	None	None
MAF100	Immersed in 46% hydrofluoric acid at DC and constant voltage of 100 V	30s
MAF150	Immersed in 46% hydrofluoric acid at DC and constant voltage of 150 V	30s
MAF200	Immersed in 46% hydrofluoric acid at DC and constant voltage of 200 V	30s
MAF250	Immersed in 46% hydrofluoric acid at DC and constant voltage of 250 V	30s
MAF300	Immersed in 46% hydrofluoric acid at DC and constant voltage of 300 V	30s

**Table 3:** Surface roughness of AZ31, MAF100, MAF150, MAF200, MAF250, and MAF300.

Code	Ra (*μ*m)	Rp (*μ*m)	Rq (*μ*m)	Rt (*μ*m)	Rv (*μ*m)
AZ31	0.182	1.670	0.229	2.800	-1.130
MAF100	0.219	1.346	0.286	4.538	-3.191
MAF150	0.294	2.359	0.381	4.840	-2.481
MAF200	2.010	8.409	2.457	17.213	-8.804
MAF250	3.235	7.090	4.490	30.260	-23.170
MAF300	6.689	30.095	8.608	53.162	-23.067

**Table 4:** Values (mean ± SD) of contact angel and energy for Ti, AZ31, MAF100, MAF150, MAF200, MAF250, and MAF300.

Code	Contact angle (°)		E (mJ/m^2^)		
	Deionized water	Di-iodomethane	*γ* ^*d*^_*s*_^∗^	*γ* ^*p*^_*s*_^∗^	SFE
Ti	64.5 ± 12.9	51.1 ± 3.8	33.67 ± 0.65	12.87 ± 6.09	46.55 ± 6.55
AZ31	24.4 ± 2.4	33.0 ± 4.4	42.85 ± 1.68	29.80 ± 0.55	72.65 ± 1.42
MAF100	24.4 ± 2.4	147.0 ± 15.1	0.57 ± 0.61	46.91 ± 13.18	47.48 ± 12.58
MAF150	57.4 ± 17.2	104.6 ± 44.1	12.56 ± 15.00	39.31 ± 27.14	51.87 ± 19.82
MAF200	32.9 ± 14.5	46.4 ± 1.8	36.27 ± 0.56	28.46 ± 6.60	64.73 ± 6.74
MAF250	15.6 ± 8.7	24.5 ± 6.5	46.29 ± 1.95	30.56 ± 1.13	76.86 ± 3.08
MAF300	20.7 ± 13.5	46.4 ± 10.3	36.03 ± 5.26	34.26 ± 6.74	70.29 ± 6.20

^∗^ *γ* ^*d*^_*s*_ and *γ*^*p*^_*s*_ refer to dipole-dipole interactions and dispersion force components, respectively.

**Table 5:** Corrosion potential and current densities of AZ31, MAF100, MAF150, MAF200, MAF250, and MAF300.

Code	*E*_0_ (*V*)	*I*_corr_ (*μ*A/cm^2^)
AZ31	-1.501	342.4
MAF100	-1.336	0.295
MAF150	-1.318	0.228
MAF200	-1.262	0.177
MAF250	-1.293	0.199
MAF300	-1.328	0.212

## Data Availability

Data used to support findings of this study are included within the article.
